# Diagnostic Performance of Plasma SP-D, KL-6, and CC16 in Acutely Hospitalised Patients Suspected of Having Community-Acquired Pneumonia—A Diagnostic Accuracy Study

**DOI:** 10.3390/diagnostics14121283

**Published:** 2024-06-17

**Authors:** Anne Heltborg, Christian B. Mogensen, Eline S. Andersen, Mariana B. Cartuliares, Eva R. B. Petersen, Thor A. Skovsted, Stefan Posth, Ole Graumann, Morten J. Lorentzen, Mathias A. Hertz, Claus L. Brasen, Helene Skjøt-Arkil

**Affiliations:** 1 Department of Emergency Medicine, University Hospital of Southern Denmark, 6200 Aabenraa, Denmarkhsa@rsyd.dk (H.S.-A.); 2 Department of Regional Health Research, University of Southern Denmark, 5230 Odense, Denmark; 3 Department of Biochemistry and Immunology, Lillebaelt Hospital, University Hospital of Southern Denmark, 7100 Vejle, Denmark; 4 Department of Blood Tests, Biochemistry and Immunology, University Hospital of Southern Denmark, 6200 Aabenraa, Denmark; 5 Department of Clinical Research, University of Southern Denmark, 5230 Odense, Denmark; 6 Department of Emergency Medicine, Odense University Hospital, 5000 Odense, Denmark; 7 Department of Radiology, Aarhus University Hospital, 8200 Aarhus, Denmark; 8 Department of Clinical Medicine, Aarhus University, 8200 Aarhus, Denmark; 9 Department of Infectious Diseases, Odense University Hospital, 5000 Odense, Denmark

**Keywords:** community-acquired pneumonia, uteroglobin, surfactant protein-D, KL-6, diagnosis, prognosis, emergency medicine

## Abstract

Community-acquired pneumonia is a common cause of acute hospitalisation. Identifying patients with community-acquired pneumonia among patients suspected of having the disease can be a challenge, which causes unnecessary antibiotic treatment. We investigated whether the circulatory pulmonary injury markers surfactant protein D (SP-D), Krebs von den Lungen-6 (KL-6), and Club cell protein 16 (CC16) could help identify patients with community-acquired pneumonia upon acute admission. In this multi-centre diagnostic accuracy study, SP-D, KL-6, and CC16 were quantified in plasma samples from acutely hospitalised patients with provisional diagnoses of community-acquired pneumonia. The area under the receiver operator characteristics curve (AUC) was calculated for each marker against the following outcomes: patients’ final diagnoses regarding community-acquired pneumonia assigned by an expert panel, and pneumonic findings on chest CTs. Plasma samples from 339 patients were analysed. The prevalence of community-acquired pneumonia was 63%. AUCs for each marker against both final diagnoses and chest CT diagnoses ranged between 0.50 and 0.56. Thus, SP-D, KL-6, and CC16 demonstrated poor diagnostic performance for community-acquired pneumonia in acutely hospitalised patients. Our findings indicate that the markers cannot readily assist physicians in confirming or ruling out community-acquired pneumonia.

## 1. Introduction

Community-acquired pneumonia (CAP) belongs to the group of communicable diseases causing most deaths worldwide [1]. Diagnosing and treating patients suspected of having CAP is an everyday practice in emergency departments. Confirming or ruling out CAP early upon hospital arrival poses diagnostic challenges, as does identifying the aetiology in patients with CAP [2,3,4,5], and the problem of unnecessary treatment with antibiotics to target CAP is substantial [6,7]. Hence, there is a need for enhanced upfront diagnostic tools for suspected CAP patients.

CAP is an infection in the pulmonary tissue acquired outside the hospital causing a local and systemic response. Classically, diagnosis is based on a varying combination of typical symptoms (e.g., cough, purulent expectoration, dyspnoea, and fever), findings (e.g., tachypnoea and abnormal chest stethoscopy), and radiological identification of a new pulmonary infiltrate [4,8]. Circulatory biomarkers like C-reactive protein (CRP), white blood cell count, and procalcitonin can support diagnosis [9].

Most research on circulatory biomarkers for diagnosing CAP has focused on markers of the systemic inflammatory response [9]. These markers are unspecific and have limited discriminative abilities. Studies on circulatory biomarkers representing pulmonary injury have been conducted in chronic pulmonary diseases and acute respiratory distress syndrome [10,11]. Some of the pulmonary injury markers of relevance for acute injury are surfactant protein D (SP-D), Krebs von den Lungen-6 (KL-6), and Club cell protein 16 (CC16) [12].

SP-D is a collectin produced by the pulmonary epithelial cells. Translocation to the circulation occurs due to loss of integrity of the air–blood barrier. Hereby, circulatory levels can serve as a biomarker for pulmonary injury [13]. SP-D is lower on the day of admission in acutely hospitalised patients with CAP compared to healthy controls [14,15], and higher SP-D values are related to worse prognoses [16].

KL-6 is another pulmonary injury marker, mainly expressed by damaged type 2 pneumocytes. Circulating values help in the diagnosis and management of interstitial lung diseases [17]. KL-6 has been subject to much COVID-19 pneumonia research and seems to be a useful marker for disease severity and prognosis [18,19].

CC16 is a member of the secretoglobin protein family. It is reported under various names, e.g., uteroglobin and CC10 [20]. A main site of production is the non-ciliated club cells in the bronchiole epithelia. Serum values are linked to both acute and chronic pulmonary injury [21]. 

Research in CAP is generally scarce regarding the diagnostic abilities of the above markers of pulmonary injury, and their ability to differentiate patients with a provisional CAP diagnosis is still being determined. We hypothesised that they could assist in the differentiation of acutely hospitalised patients suspected of having CAP, which could be of valuable assistance in targeting CAP treatment in hospitals.

The aim was to explore the diagnostic performance of SP-D, KL-6, and CC16 in acute hospitalised patients clinically suspected of having CAP. Further, we aimed to investigate how SP-D, KL-6, and CC16 relate to diagnostic CT findings, aetiology, illness duration, and 30-day mortality in patients with a final diagnosis of CAP.

## 2. Materials and Methods

### 2.1. Study Design and Setting

This was a pragmatic, multi-centre, explorative diagnostic accuracy study with prospective data collection. The study was part of a larger collaboration project on improving the diagnosis of infectious diseases in emergency departments [22]. The present study, including the selection of the biomarkers, was conducted through a collaboration between clinical specialists and specialists in clinical biochemistry. The reporting was guided by the Standard for Reporting Diagnostic Accuracy Studies (STARD) statement [23]. 

Patient inclusion was conducted by six study assistants in the acute medicine unit of three Danish hospitals between March 2021 and February 2022. The population was a convenience sample; patients were included continuously in the presence of the study assistants during the inclusion period, which was weekdays between 8 a.m. and 8 p.m.

### 2.2. Study Population

Participants were acutely hospitalised adults (≥18 years old); patients were eligible if the receiving physician suspected the presence of CAP upon an initial clinical assessment. Within the first few hours of arrival, eligible patients were included if they did not meet any of the following exclusion criteria: admission within the last 14 days (to avoid hospital-acquired infections), undergoing immunosuppressive treatment, incapability or unwillingness to provide informed consent to participate, pregnancy, capacity limitations faced by the study assistants, or critically ill and requiring urgent, life-saving treatment based on the receiving physicians’ judgement. Further, we excluded patients with a new, positive SARS-CoV-2 test from within the last 14 days, to avoid coronavirus disease 2019 dominance in the study population.

### 2.3. Plasma Sample Procedure

Venous blood samples were collected by phlebotomists or trained study assistants in BD Vacutainer^®^ Lithium Heparin and ethylenediaminetetraacetic acid (EDTA) tubes (Becton, Dickinson and Company, Franklin Lakes, NJ, USA) immediately upon patient inclusion. The samples were centrifuged, and plasma was pipetted into cryo tubes and stored at minimum −20 °C within two hours from the blood draw. Samples from all inclusion sites were transported, frozen, to a single laboratory and kept frozen until being collectively analysed after completion of patient inclusion.

### 2.4. Biomarker Analyses—Index Tests

All analyses were conducted in the Department of Biochemistry and Immunology at a Danish Hospital (Lillebaelt Hospital, Vejle, Denmark). Experienced biomedical laboratory scientists ran the analyses according to the manufacturer’s instructions.

We used enzyme-linked immunosorbent assay (ELISA)-based analysis kits. KL-6 was quantified using Nanopia^®^ KL-6 Reagent (Sekisui Diagnostic, Burlington, MA, USA) and EDTA plasma on a Roche cobas c502 module. SP-D was quantified using Quantikine™ Human SP-D Immunoassay (R&D Systems, Inc., Minneapolis, MN, USA) and Lithium Heparin plasma. CC16 was quantified using Quantikine^®^ Human Uteroglobin Immunoassay (R&D Systems, Inc., Minneapolis, MN, USA) and EDTA plasma. SP-D and CC16 were analysed manually. KL-6 was analysed in the first freeze/thaw cycle, and CC16 and SP-D in the second cycle.

### 2.5. Reference Standard

The primary reference standard comprised the participants’ final diagnoses, as assigned by two medical experts. One emergency medicine consultant and one infectious diseases consultant independently assigned a final diagnosis to each participant. The final diagnoses were based on expert opinion from chart reviews using all available data, including imaging and standard laboratory results from at least one week after inclusion. No checklists or diagnostic criteria were applied. Disagreements in the final diagnoses were resolved by the two assigning experts in collaboration. In total, eight experts were involved in the study. They were blinded to the pulmonary injury biomarker results. We categorised all final diagnoses into CAP or no CAP for the analyses.

A standard-dose computed tomography (CT) of the chest was available from most participants (82%). These images and their reports were also available to the experts. Further, CTs were read by a radiologist with expertise in thoracic CTs, and the presence of changes corresponding to pneumonia was used as reference standard in secondary analyses. The presence of CAP changes was based on the radiologist’s identification of consolidations that were not in a tumour or nodular pattern, ground-glass opacities, tree-in-bud patterns, or poorly defined per-bronchial nodules observed in bronchopneumonia.

### 2.6. Other Variables

Baseline characteristics, routine biomarker results, lung auscultation results, vitals and triage at arrival (based on Danish Emergency Process Triage [24]), and follow-up data on 30-day mortality were collected from the patients’ medical records. Information on current symptoms and symptom onset was collected from patient interviews. All data were registered in an online data collection tool (Research Electronic Data Capture, REDCap, versions 10.8.3–12.2.1, Vanderbilt University, Nashville, TN, USA).

Aetiological testing with point-of-care polymerase chain reaction (PCR) analysis of a sputum sample was available for a sub-population of CAP patients using Biofire FilmArray Pneumonia Panel plus (Biomérieux, Marcy l’Etoile, France) [25]. Results were categorised into bacterial, viral, no detection, or mixed bacterial and viral findings and then compared to the biomarkers.

### 2.7. Statistics

Patient characteristics were summarised with descriptive statistics using median and interquartile ranges for continuous variables, and numbers and percentages for categorical variables. Medians were compared using Kruskal–Wallis tests. The diagnostic performance of the biomarkers was expressed as the area under the curve (AUC) of non-parametric receiver operating characteristic (ROC) curves. To investigate whether the biomarkers added diagnostic value when combined with known predictors of CAP, we created a baseline logistic regression model of established predictors. We used categorical predictors as identified in a recent study using part of the same study population [26]. AUCs were calculated from the predicted values with and without incorporating the biomarkers into the model. A graphical overview of median biomarker levels in relation to symptom duration was conducted to assess the impact of acute illness duration on biomarker levels. Complete case analyses were performed.

The sample size for this study was not based on pre-specified power calculations; we used all available data from the overall project. Data management and analyses were performed using Stata/BE version 17.0 (StataCorp LLC, College Station, TX, USA).

## 3. Results

### 3.1. Study Population

**Figure 1:**
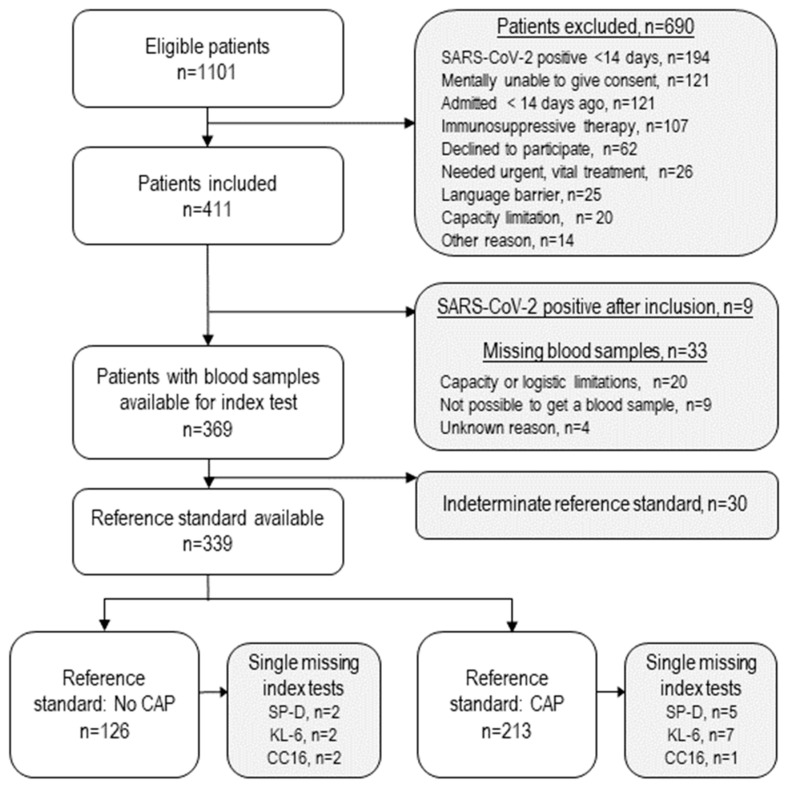
Flowchart of participants and causes for exclusion. CAP: community-acquired pneumonia.

This study included 339 patients in the analyses (Figure 1). Thirty patients’ final diagnoses were considered indeterminate for the CAP/no-CAP categorisation. They were diagnosed with infection-induced exacerbation of chronic obstructive pulmonary disease, but the location of the infection was unclear. Thus, they were excluded from the analyses. The reasons for missing blood samples or biomarker results were mainly capacity or logistical limitations. Unsuccessful attempts to collect blood caused missing samples for nine patients.

The prevalence of patients with a final diagnosis of CAP in the study population was 63%. Of the 126 patients without CAP, 62 (49%) had another infection, and 64 (51%) were not infected but suffered from a variety of other conditions, including chronic obstructive pulmonary disease (n = 17), and pulmonary embolism (n = 3). Sixteen patients had final diagnoses of unspecific symptoms: either dyspnoea, cough, or chest pain. Further characteristics of the study population are summarised in Table 1.

### 3.2. Diagnostic Performance

**Figure 2:**
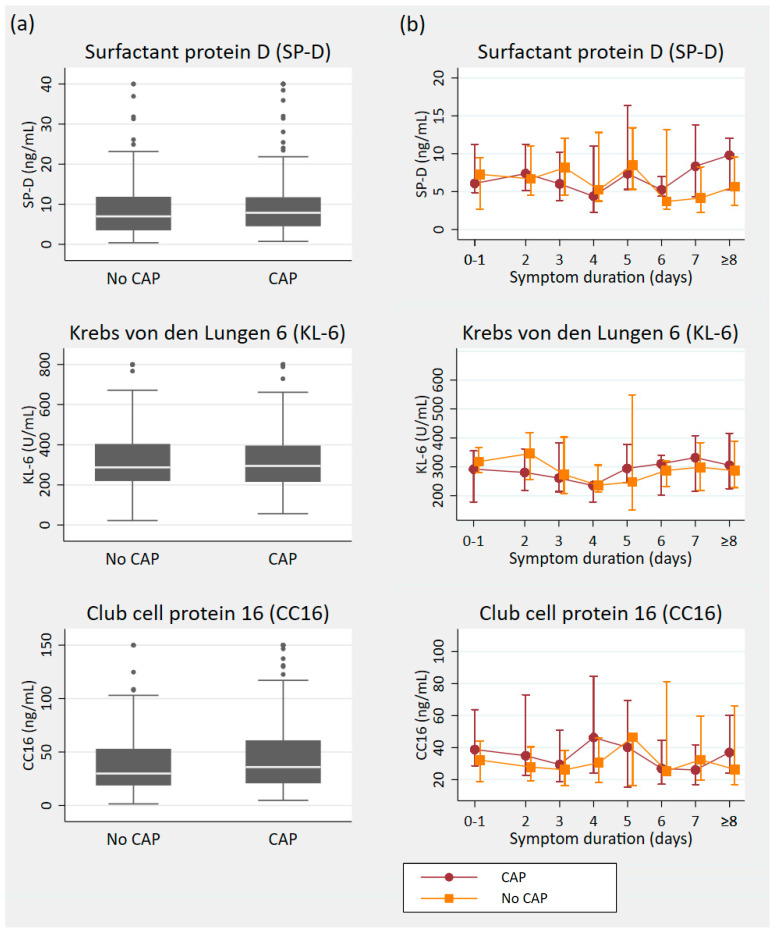
(**a**) Box plot summary of the pulmonary injury markers by community-acquired pneumonia (CAP) diagnosis. Eight SP-D results > 40 ng/mL (max. 123 ng/mL) were collapsed to 40 ng/mL (six CAP cases), sixteen KL-6 results > 800 U/mL (max. 3916 U/mL) were collapsed to 800 U/mL (11 CAP cases), and eleven CC16 results > 150 ng/mL (max. 610 ng/mL) were collapsed to 150 ng/mL (eight CAP cases). (**b**) Median biomarker levels with interquartile ranges in relation to symptom duration. CAP: community-acquired pneumonia.

Figure 2a depicts the overall similar levels of each pulmonary injury marker in patients with and without CAP. Table 2 presents the results of the diagnostic performance of SP-D, KL-6, and CC16 in CAP. The AUCs for all biomarkers are close to 0.5 when compared to the final diagnoses. As expected from these results, incorporating the biomarkers into the baseline model of known predictors does not improve the model’s AUC. The comparison of biomarkers with CAP-related changes on chest CTs yielded AUC values ranging from 0.46 to 0.6.

Considering the biomarkers in relation to symptom duration did not reveal a diagnostically valuable pattern (Figure 2b). The median values followed a similar trajectory, and all interquartile ranges exhibited large overlaps.

### 3.3. Aetiology

PCR results from a sputum sample were available for 85 patients with a final diagnosis of CAP. Most results were bacterial (n = 45). Fewer were mixed bacterial and viral (n = 18), viral only (n = 6), or negative (n = 16). No intracellular bacteria were detected in this population. Median biomarker values generally tended to be higher in patients with only viral detection, but no evidence of statistical difference was reached in these small numbers of cases (Table 3).

### 3.4. Mortality

In total, 11 (5%) of the 213 patients with a final diagnosis of CAP died within 30 days of study inclusion. SP-D, KL-6, and CC16 were all higher in the deceased compared to survivors after 30 days (Table 4). KL-6 demonstrated the greatest discriminative ability, with an AUC of 0.90 (95%CI: 0.85–0.95).

## 4. Discussion

This study demonstrated a poor diagnostic performance of the pulmonary injury biomarkers SP-D, KL-6, and CC16 for diagnosing CAP in acutely hospitalised patients initially suspected of having CAP by the receiving physician. The lack of classification abilities for all three markers was evident with AUCs between 0.50 and 0.56 against both the final diagnoses and chest CTs. We identified no significant associations between the biomarker values and microbiological findings in a small sub-population with PCR analyses on available sputum samples, and no association with illness duration. Our results indicate that these biomarkers cannot assist physicians in differentiating patients with a provisional CAP diagnosis in the acute setting. However, all three biomarkers indicated prognostic abilities concerning 30-day mortality, with KL-6 demonstrating the highest AUC of 0.90 (95%CI: 0.85–0.95).

To our knowledge, this is the first study challenging the diagnostic and prognostic performance of SP-D, KL-6, and CC16 for CAP in a heterogeneous emergency department population suspected of having CAP. The course of SP-D has previously been investigated in acutely hospitalised patients with verified CAP, with findings of generally lower levels of SP-D on the day of admission compared to healthy controls and rising values across the days following admission [14,15]. A similar tendency of lower SP-D on admission day did not occur in our study, which might be due to both the non-healthy patients we used for comparison, and differences in the CAP populations. The other studies’ CAP populations were selected based on diagnostic criteria, including a new infiltrate on chest X-ray [14,15]. These criteria probably excluded some patients with early-stage or otherwise radiographically unidentifiable pneumonia [27], who were included in our population. The early-stage CAP patients could challenge our hypothesis if the extend of acute lung damage was not sufficient to cause relevant changes in potential pulmonary injury biomarkers. Yet, the biomarkers did not exhibit a temporal pattern in our investigation, suggesting that illness duration alone does not influence biomarker levels.

SP-D and CC16 values have previously been compared with the aetiology of CAP, with no findings of differing values for viruses and bacteria [15,28]. Contrary to our findings, SP-D was non-significantly lower in 19 patients with viral CAP compared to bacterial aetiologies [15], suggesting no substantial difference.

Despite there being few deaths in our relatively low-risk population, the association between higher biomarker values and 30-day mortality is remarkable. KL-6 revealed the most robust prognostic performance in this case. This finding aligns with findings in COVID-19 pneumonia and severe CAP [19,29]. In opposition to our findings, CC16 was lower in CAP patients with increased mortality risk in a Chinese study population [28].

A strength of this study was the certainty of the patients’ final diagnoses obtained by dual chart review by medical experts. The decision not to use checklists or criteria to perform the final diagnoses was made in line with this study’s pragmatic approach, as a CAP diagnosis is based on the sum of many individual parts [4]. Other strengths were the high availability of chest CTs and the multi-centre design.

The limitations of our study included the relatively small population with comprehensive microbiological testing, and a pathogen only being detected in some CAP patients. Further, it was not established whether the detected microorganisms were the actual causative pathogens, and if so, which of them were. Hereby, these analyses suffered from a lack of power, and we cannot draw definitive conclusions. Difficulties in defining aetiology are a general problem in CAP research [30], but need to remain a continuously high priority to obtain precise CAP diagnoses and promote targeted CAP treatment.

The applied exclusion criteria prevented the participation of the most severely ill patients in whom vital treatment could not be delayed by study participation. This criterion resulted in the exclusion of 26 patients and potential selection bias. Our study prioritised the less critically urgent patients, as they are the most common patients in the emergency departments, and their cases offer more time for diagnostics before antibiotic treatment [31]. Yet, these results cannot be extrapolated to the very most severely ill patients with pneumonia.

The pulmonary injury biomarkers generally exhibit the strongest association with pneumonia severity [16,32], potentially contributing to the ambiguous biomarker pattern observed in our investigation, compared to both final diagnoses and CTs. Previously, an association between higher KL-6 and the presence of ground-glass opacities was reported in patients with severe CAP [29], which was not present in our study. Probably, the general magnitude of pathological pulmonary changes is more significant in a population with severe CAP compared to our more mixed population. To account for this limitation, we could have investigated the extent of acute pulmonary changes on the CT scans. However, this was not a part of our original CT readings, and we did not have access to a standardised tool for subsequently quantifying lung injury.

The statistical analyses used in this study were relatively simple, and no linear association with the outcome appeared from the crude biomarker values. More advanced machine learning or artificial intelligence methods might capture more complex patterns in the biomarkers and factors of importance for constitutional levels in each patient in relation to a CAP diagnosis. Our study population was limited by being relatively small for these types of analyses [33], and the study aimed to investigate a more pragmatic utility of the markers. Yet, if any of the markers is considered relevant for this approach in the future, data from this study could be incorporated.

In conclusion, our investigation reveals no utility of plasma SP-D, KL-6, or CC16 for diagnosing CAP in acutely hospitalised patients with CAP as a provisional diagnosis. We cannot exclude the ability of these pulmonary injury biomarkers to contribute to more advanced diagnostic models, but they are not candidates for rapid and intuitive diagnostic use in the clinical setting. All markers demonstrated prognostic abilities for short-term mortality in patients with CAP, especially KL-6. More extensive studies with higher numbers of severe outcomes, including death, could clarify whether the markers could improve the risk stratification of patients with CAP.

## Figures and Tables

**Table 1:** Description of the study population.

	Total (n = 339)	No CAP (n = 126)	CAP (n = 213)	Missing, Total (No CAP/CAP)
Male sex	185 (55%)	73 (58%)	112 (53%)	
Age (years)	74 (62–82)	73 (59–81)	74 (64–82)	
Antibiotics before hospitalisation	86 (25%)	22 (17%)	64 (30%)	
Comorbidities and risk factors				
Chronic obstructive pulmonary disease	103 (30%)	39 (31%)	64 (30%)	
Congestive heart failure	30 (9%)	12 (10%)	18 (8%)	
Ischaemic heart disease	55 (16%)	25 (20%)	30 (14%)	
Type 2 diabetes	50 (15%)	18 (14%)	32 (15%)	
Chronic kidney disease	18 (5%)	7 (6%)	11 (5%)	
Current smoker	73 (22%)	29 (24%)	44 (21%)	14 (6/8)
Vital signs and triage at arrival				
Peripheral oxygen saturation (%)	95 (93–97)	96 (94–98)	95 (93–97)	1 (1/0)
Respiratory rate pr. minute	20 (18–24)	20 (18–24)	21 (18–24)	1 (0/1)
Temperature (°C)	37.3 (36.8–38)	37.1 (36.6–37.8)	37.5 (36.8–38.2)	2 (0/2)
Triage * red or orange	103 (33%)	34 (30%)	69 (34%)	25 (13/12)
Symptoms ▪				
Cough	236 (72%)	78 (65%)	158 (77%)	13 (6/7)
Increased expectoration	178 (55%)	55 (46%)	123 (60%)	13 (6/7)
Dyspnoea	232 (71%)	84 (70%)	148 (72%)	13 (6/7)
Fever/fever sensation †	161 (47%)	57 (45%)	104 (49%)	
Malaise	208 (64%)	71 (60%)	137 (67%)	16 (8/8)
Symptom duration (days) ‡	6 (3–9)	6 (3–10)	6 (3–9)	27 (11/16)
Inflammatory biomarkers				
C-reactive protein (mg/L)	108 (40–193)	50 (11–142)	135 (68–212)	
White blood cell count (109/L)	11.4 (8.4–15.2)	9.1 (7.2–12.8)	12.3 (9.7–16)	
Procalcitonin (µg/L)	0.14 (0.06–0.48)	0.095 (0.03–0.39)	0.17 (0.07–0.6)	1 (0/1)
Chest computed tomography (CT)				
CAP findings present ♦	166 (59%)	25 (26%)	141 (77%)	60 (30/30)
Consolidation, pneumonic	111 (40%)	10 (10%)	101 (55%)	60 (30/30)
Ground-glass opacity	72 (26%)	15 (16%)	57 (31%)	60 (30/30)
Tree-in-bud pattern	92 (33%)	11 (11%)	81 (44%)	60 (30/30)

CAP: community-acquired pneumonia. Values are presented either as n (%) or median (interquartile range) for Total, CAP, and No CAP. * Danish Emergency Process Triage [24] defines 5 urgency levels based on vitals on arrival and suspected condition, with red and orange being most urgent. **▪** Patient-reported. Present symptoms with new onset or worsening within the last 14 days were registered. † Fever measured at home, or fever symptoms (night sweat or chills). ‡ Earliest onset date of any new symptom reported by the patient. ♦ Overall assessment by a radiologist with expertise in thoracic CTs.

**Table 2:** Diagnostic performance of SP-D, KL-6, and CC16 in patients suspected of having CAP.

Reference: Final Diagnosis (n = 339)			
	Biomarker	AUC	95%CI
Univariate analyses	SP-D	0.55	0.48–0.61
	KL-6	0.50	0.43–0.57
	CC16	0.53	0.47–0.59
Biomarkers added to a baseline model of known CAP predictors *	Baseline model	0.80	0.74–0.85
	Baseline model + SP-D	0.80	0.74–0.85
	Baseline model + KL-6	0.81	0.75–0.86
	Baseline model + CC16	0.80	0.74–0.85
**Reference: Findings on Chest CTs (n = 279)**			
**CT Finding**	**Biomarker**	**AUC**	**95%CI**
Community-acquired pneumonia ▪	SP-D	0.56	0.50–0.63
	KL-6	0.50	0.43–0.57
	CC16	0.51	0.44–0.58
Consolidation, pneumonia pattern	SP-D	0.55	0.48–0.62
	KL-6	0.51	0.44–0.58
	CC16	0.54	0.48–0.61
Ground-glass opacity	SP-D	0.60	0.52–0.69
	KL-6	0.59	0.51–0.66
	CC16	0.60	0.52–0.67
Tree-in-bud pattern	SP-D	0.55	0.48–0.62
	KL-6	0.46	0.39–0.53
	CC16	0.46	0.39–0.53

CAP: community-acquired pneumonia. * Predictors included: dyspnoea, expectoration, cough, cold, malaise, chest pain, respiratory rate > 20/min, oxygen saturation < 96%, abnormal lung auscultation, abnormal white blood cell count, elevated neutrophilocytes, and C-reactive protein < 20 mg/L [26]. ▪ Overall assessment based on the identification of consolidations that were not in a tumour or nodular pattern, ground-glass opacities, tree-in-bud patterns, or poorly defined per-bronchial nodules.

**Table 3:** SP-D, KL-6, and CC16 values in patients with community-acquired pneumonia by microorganisms detected in PCR analyses of sputum samples.

Biomarker	Bacterial (n = 45)	Viral (n = 6)	Bacterial and Viral (n = 18)	No Microorganisms (n = 16)	*p*-Value
SP-D, ng/mL	6.5 (3.6–11.2)	10.1 (5.3–14)	5.3 (4.5–11.4)	5.5 (3.6–10.5)	0.76
KL-6, U/mL	281 (214–382)	392 (323–411)	256 (210–320)	317 (213–383)	0.34
CC16, ng/mL	33 (18–67)	53 (26–78)	22 (19–53)	30 (24–51)	0.36

Biomarker values are presented as median (interquartile range).

**Table 4:** SP-D, KL-6, and CC16 compared to 30-day mortality in patients with community-acquired pneumonia.

Biomarker	Alive (n = 202)	Deceased (n = 11)	Missing Values	*p*-Value	AUC (95%CI)
SP-D, ng/mL	7.4 (4.5–11.4)	11.6 (8–36)	1/1	0.025	0.71 (0.54–0.88)
KL-6, U/mL	285 (214–377)	513 (429–641)	1/1	<0.001	0.90 (0.85–0.95)
CC16, ng/mL	34 (21–59)	64 (25–192)	2/0	0.016	0.72 (0.53–0.90)

Biomarker values are presented as median (interquartile range).

## Data Availability

The data presented in this study are available on request from the corresponding author. Due to Danish laws on personal data, data cannot be shared publicly.
